# Supplementary material for: Plant Alkylbenzenes and Terpenoids in the Form of Cyclodextrin Inclusion Complexes as Antibacterial Agents and Levofloxacin Synergists
Source: Pharmaceuticals (Basel). 2022 Jul 14;15(7):861. doi: 10.3390/ph15070861 (PMC9321718; doi:10.3390/ph15070861)

Supplementary Materials

Plant alkylbenzenes and terpenoids in the form of cyclodextrin inclusion complexes as antibacterial agents and levofloxacin synergists

Igor D. Zlotnikov [1], Natalya G. Belogurova [1], Sergey S. Krylov [2], Marina N. Semenova [3], Victor V. Semenov [2] and Elena V. Kudryashova [1,*]

[1] Department of Chemistry, M.V. Lomonosov Moscow State University, Leninskie Gory, 1/11B, 119991 Moscow, Russia; zlotnikovid@my.msu.ru (I.D.Z.); nbelog@mail.ru (N.G.B.)
[2] N. D. Zelinsky Institute of Organic Chemistry RAS, 47 Leninsky Prospect, 119991 Moscow, Russia; forvard1953@yandex.ru (S.S.K.); vs@chemical-block.com (V.V.S.)
[3] N. K. Koltzov Institute of Developmental Biology RAS, 26 Vavilov Street, 119334 Moscow, Russia; ms@chemical-block.com
* Correspondence: helena_koudriachova@hotmail.com

Content

1. Figure S1. Baseline corrected UV spectra of (a) EG, 15 mM, (b) apiol, 15.8 mM, and their inclusion complexes with HPCD. Conditions are given in Materials and Methods, sections 3.3 and 3.4.
2. Figure S2. (a) λ_{max} (nm) absorbance position of eugenol (EG) allyl group in EG-HCPD complex. (b) UV spectra of apiol-HPCD presented in Figure S1b converted to the Hill plot. Theta is a fraction of the bound substance: θ = [EG-HPCD] / ([EG-HPCD] + [EG]). Aqueous solutions, 22 °C.
3. Figure S3. Entrapment efficiency (EE) of apiol (a) and eugenol (b) into (2-hydroxypropyl)-β-cyclodextrin (HPCD) in aqueous solutions at 22 °C and A_{280}. Hill plots. The EE plateau is reached at a level lower than 100% due to the partial presence of EG and apiol in dissolved form in water, not in the form of a complex with CD. However, solubility efficiency is aiming for 100%. C_0 – theoretical concentration under the assumption that 100% of the substance has dissolved, C_{max} – the maximum practically achievable concentration.
4. Figure S4. (a) FTIR spectra peak intensity at 1505 cm^{-1} for apiol and its inclusion complexes with HPCD. (b) Minimum position of second derivative ($d^2A/d\nu^2$) of FTIR spectra of eugenol and its inclusion complexes with HPCD versus HPCD concentration. (c) Data of (b) converted to the Hill plot. Theta is a fraction of the bound substance: θ = [EG-HPCD] / ([EG-HPCD] + [EG]). Aqueous solutions, 22 °C.
5. Figure S5: Second derivative ($d^2A/d\nu^2$) of FTIR spectra of eugenol and its inclusion complex with HPCD. A baseline correction was applied. Conditions are given in Materials and Methods, sections 3.4, 3.5;
6. Figure S6: (a) Micrography of the EG-HPCD solid inclusion complex of 1.3 × 1.5 mm in size. (b), (c) Corresponding FTIR spectra at 16 points selected in (a). (d) Integral intensity of 1482–1562 cm^{-1} region in solid phase FTIR spectra of eugenol-HPCD. (e) Integral intensity of 1536–1499 cm^{-1} region in solid phase FTIR spectra, normalized to the corresponding intensity of C–O–C bonds of cyclodextrin at 1020-1180 cm^{-1};
7. Figure S7: Micrographs illustrating the inclusion of eugenol (EG) in a HPCD. Dissolving oil droplets. (a) EG, (b) EG–HPCD (3.5:1), (c) EG–HPCD (1:1), (d) EG–HPCD (1:5). (e) Crystalline EG–HPCD (1:1) inclusion complex – analog (c) after evaporation of water. The molar ratio of the components is given in parentheses. Dissolving oil droplets of undissolved eugenol. Scale bar: 100 mm;
8. Figure S8: Micrographs illustrating the inclusion of apiol in a HPCD. Dissolving process. (a) Apiol, (b) Apiol–HPCD (2:1), (c) Apiol–HPCD (1:1), (d) Apiol–HPCD (1:15). The molar ratio of the components is given in parentheses. Scale bar: 100 mm;
9. Figure S9. ^1H NMR-spectra (600 MHz) of apiol in DMSO-d6 (A); MCD-apiol in D_2O, molar ratio 2:1 (B); HPCD-EG in D_2O, molar ratio 2:1 (C); HPCD-Lev-EG in D_2O, molar ratio 4:1:1 (D). Computer simulation’s structures of CD (E), MCD (F), HPCD (G) in comparison with literature X-ray data (H) [43].
10. Figure S10: *B. subtilis* growth inhibition by (a) levofloxacin–MCD (1:2), (b) eugenol–MCD (1:2). The molar ratio of the components is given in parentheses. Small bold red crosses refer to the absence of bacterial growth inhibition; large green crosses indicate the diameter of growth inhibition zone. Experimental conditions here and thereafter in Figures S10–S13: pH 7.4 (0.01 M PBS), 37 °C, 24 h of incubation. MCD: methyl-β-cyclodextrin;

11. Figure S11: *E. coli* growth inhibition by levofloxacin–MCD (C(Lev) = 0.15 μg/mL) with adjuvant menthol–MCD (the concentrations of menthol are indicated in the figure). Small bold red crosses refer to the absence of bacterial growth inhibition; large red crosses indicate the diameter of growth inhibition zone;
12. Figure S12: *E. coli* growth inhibition by levofloxacin–MCD (C(Lev) = 0.15 mg/mL) with adjuvant apiol–MCD (the concentrations of apiol are indicated in the figure). Small bold red crosses refer to the absence of bacterial growth inhibition. Black crosses indicate the diameter of growth inhibition zone;
13. Figure S13: *B. subtilis* growth inhibition by levofloxacin–MCD (C(Lev) = 0.4; 0.7 and 1.5 mg/mL) with adjuvant eugenol–MCD (the concentrations of eugenol are indicated in the figure). Small bold red crosses refer to the absence of bacterial growth inhibition. Black crosses indicate the diameter of growth inhibition zone;
14. Figure S14: *B. subtilis* growth inhibition by levofloxacin–MCD (C(Lev) = 0.4; 0.7 and 1.5 mg/mL) with adjuvant safrole–MCD (the concentrations of safrole are indicated in the figure). Small bold red crosses refer to the absence of bacterial growth inhibition. Black crosses indicate the diameter of growth inhibition zone.

Figure S1. Baseline corrected UV spectra of (a) EG, 15 mM, (b) apiol, 15.8 mM, and their inclusion complexes with HPCD. Conditions are given in Materials and Methods, sections 3.3 and 3.4.

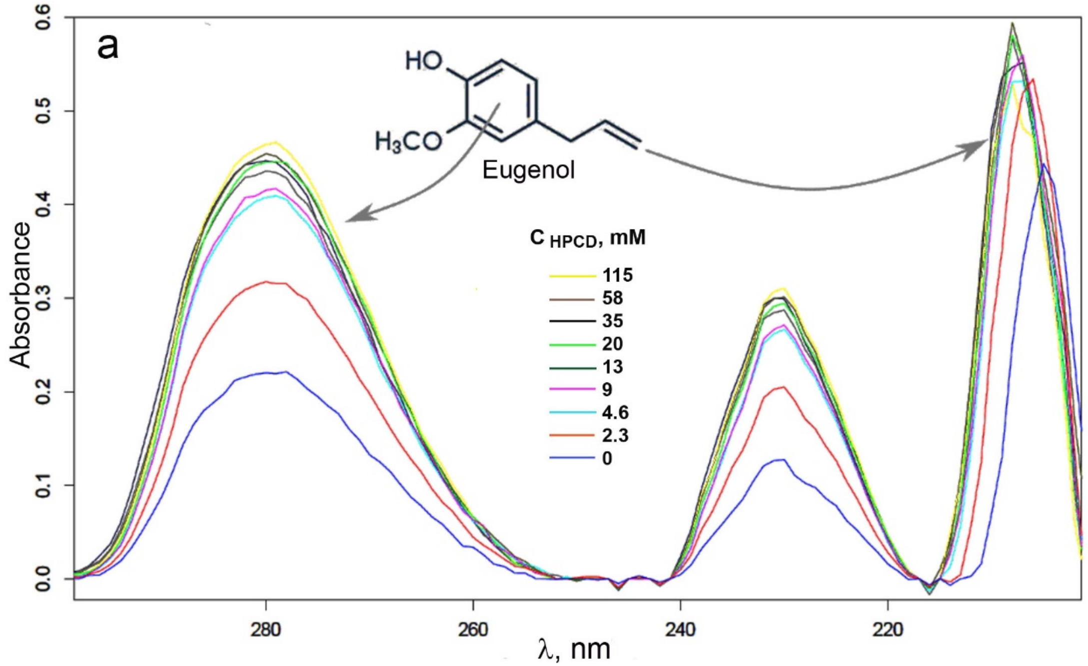

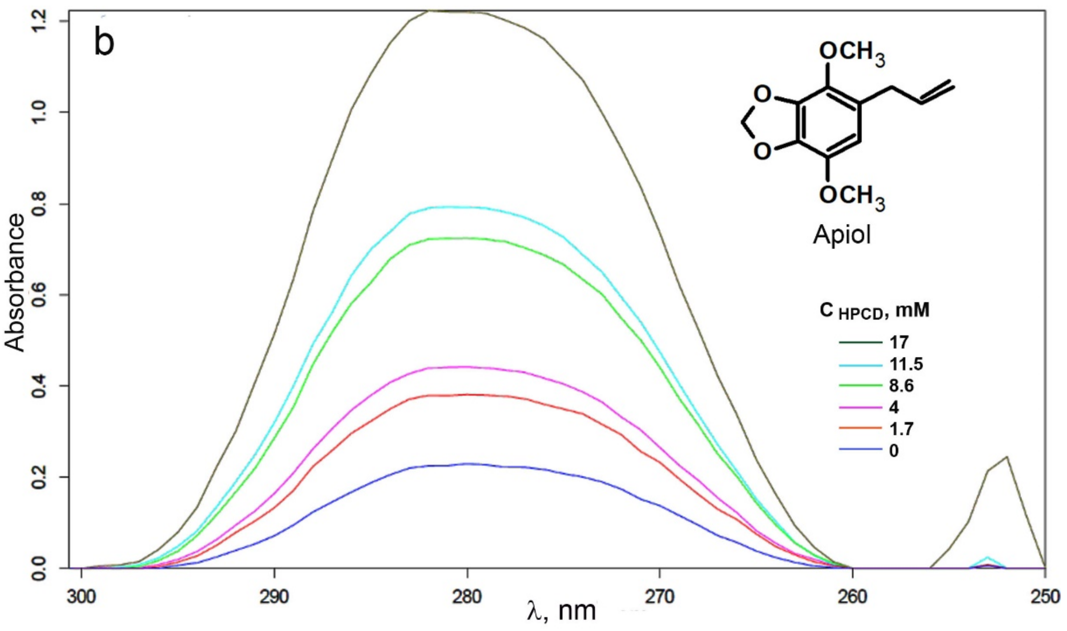
b
Absorbance
1.2
1.0
0.8
0.6
0.4
0.2
0.0
300
290
280
270
260
250
λ, nm
OCH3
O
O
OCH3
Apiol
C HPCD, mM
17
11.5
8.6
4
1.7
0

Figure S2. (a) λ_{max} (nm) absorbance position of eugenol (EG) allyl group in EG-HCPD complex. (b) UV spectra of apiol-HPCD presented in Figure S1b converted to the Hill plot. Theta is a fraction of the bound substance: θ = [EG-HPCD] / ([EG-HPCD] + [EG]). Aqueous solutions, 22 °C.

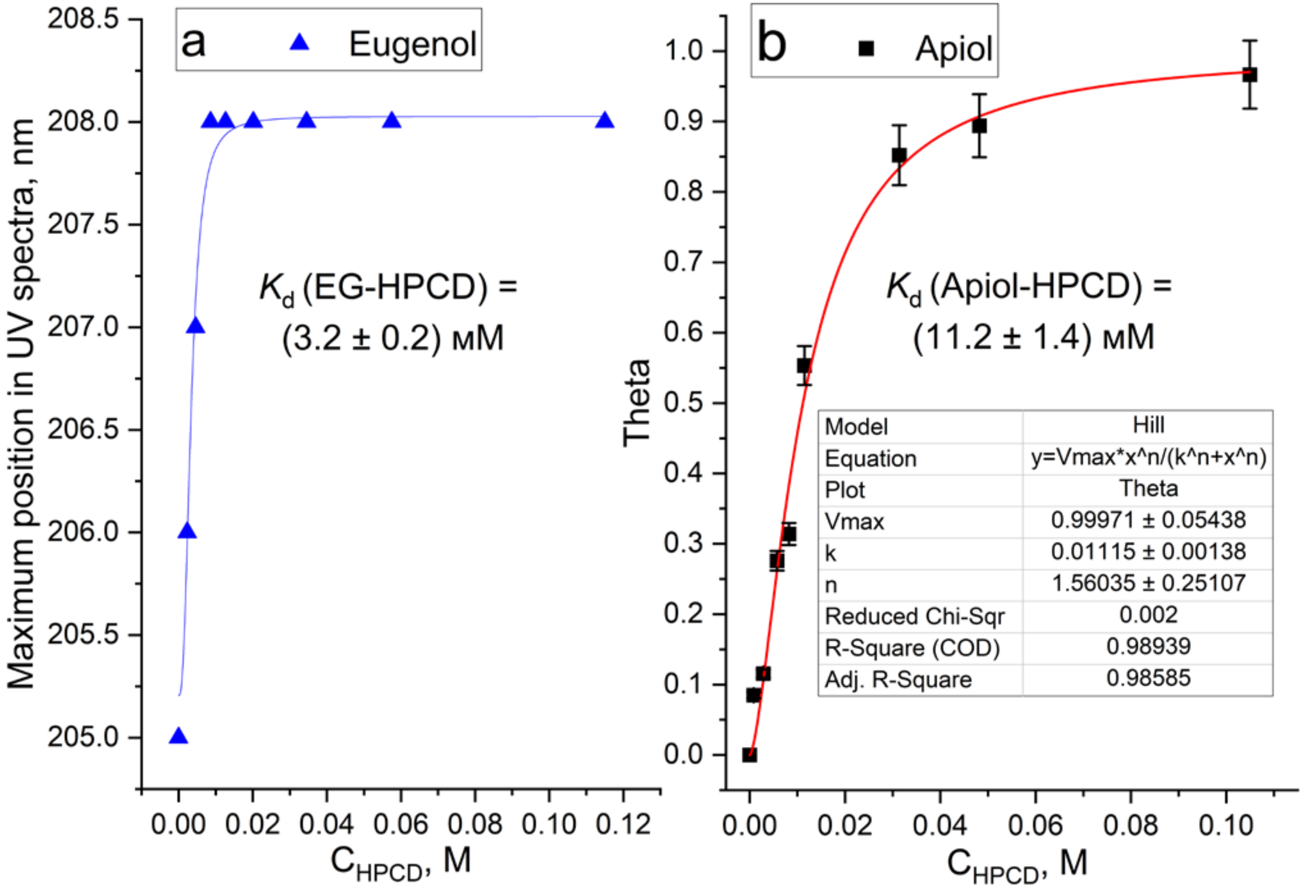

Figure S3. Entrapment efficiency (EE) of apiol (a) and eugenol (b) into (2-hydroxypropyl)-β-cyclodextrin (HPCD) in aqueous solutions at 22 °C and A_{280}. Hill plots. The EE plateau is reached at a level lower than 100% due to the partial presence of EG and apiol in dissolved form in water, not in the form of a complex with CD. However, solubility efficiency is aiming for 100%. C_0 – theoretical concentration under the assumption that 100% of the substance has dissolved, C_{max} – the maximum practically achievable concentration.

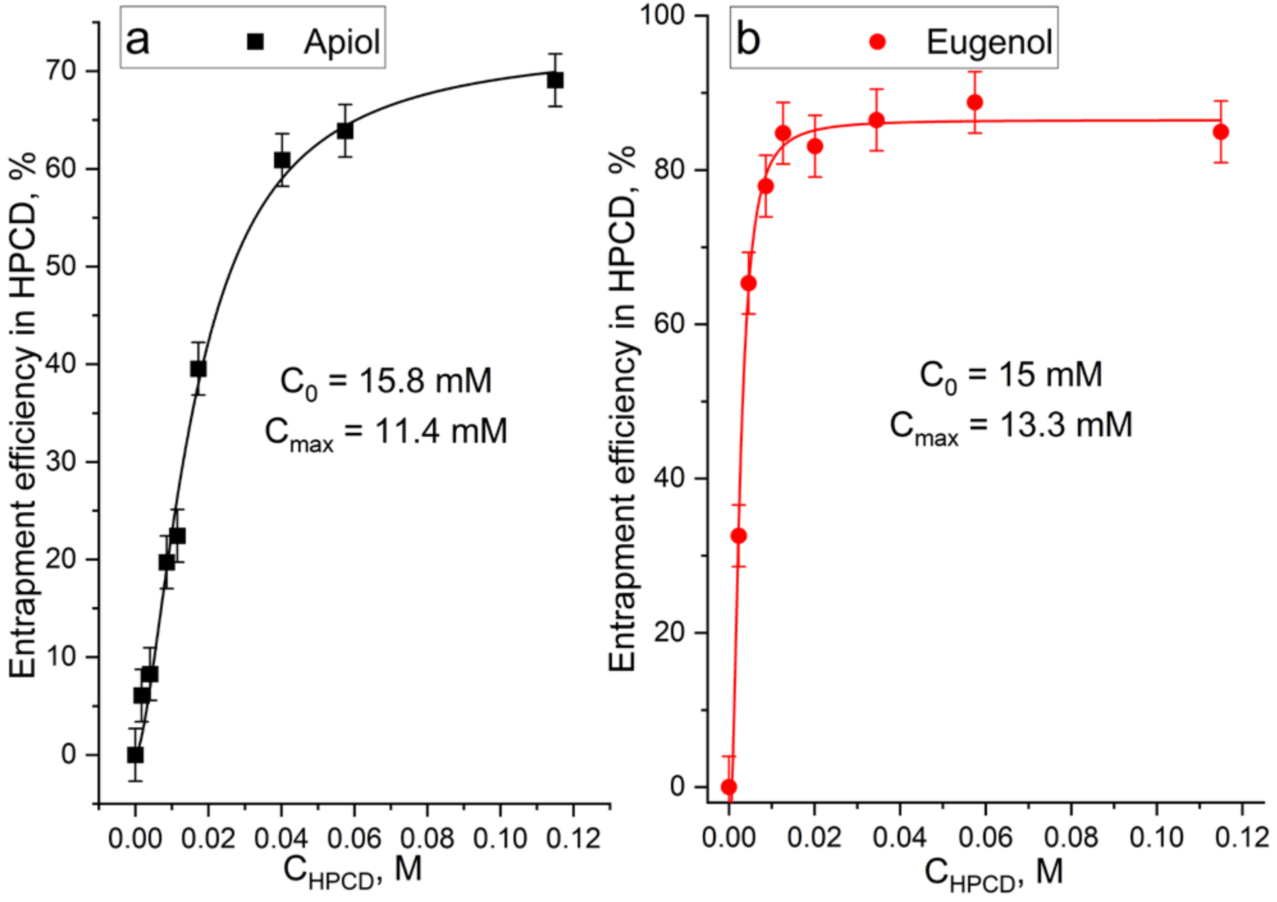

Figure S4. (a) FTIR spectra peak intensity at 1505 cm^{-1} for apiol and its inclusion complexes with HPCD. (b) Minimum position of second derivative ($d^2A/d\nu^2$) of FTIR spectra of eugenol and its inclusion complexes with HPCD versus HPCD concentration. (c) Data of (b) converted to the Hill plot. Theta is a fraction of the bound substance: θ = [EG-HPCD] / ([EG-HPCD] + [EG]). Aqueous solutions, 22 °C.

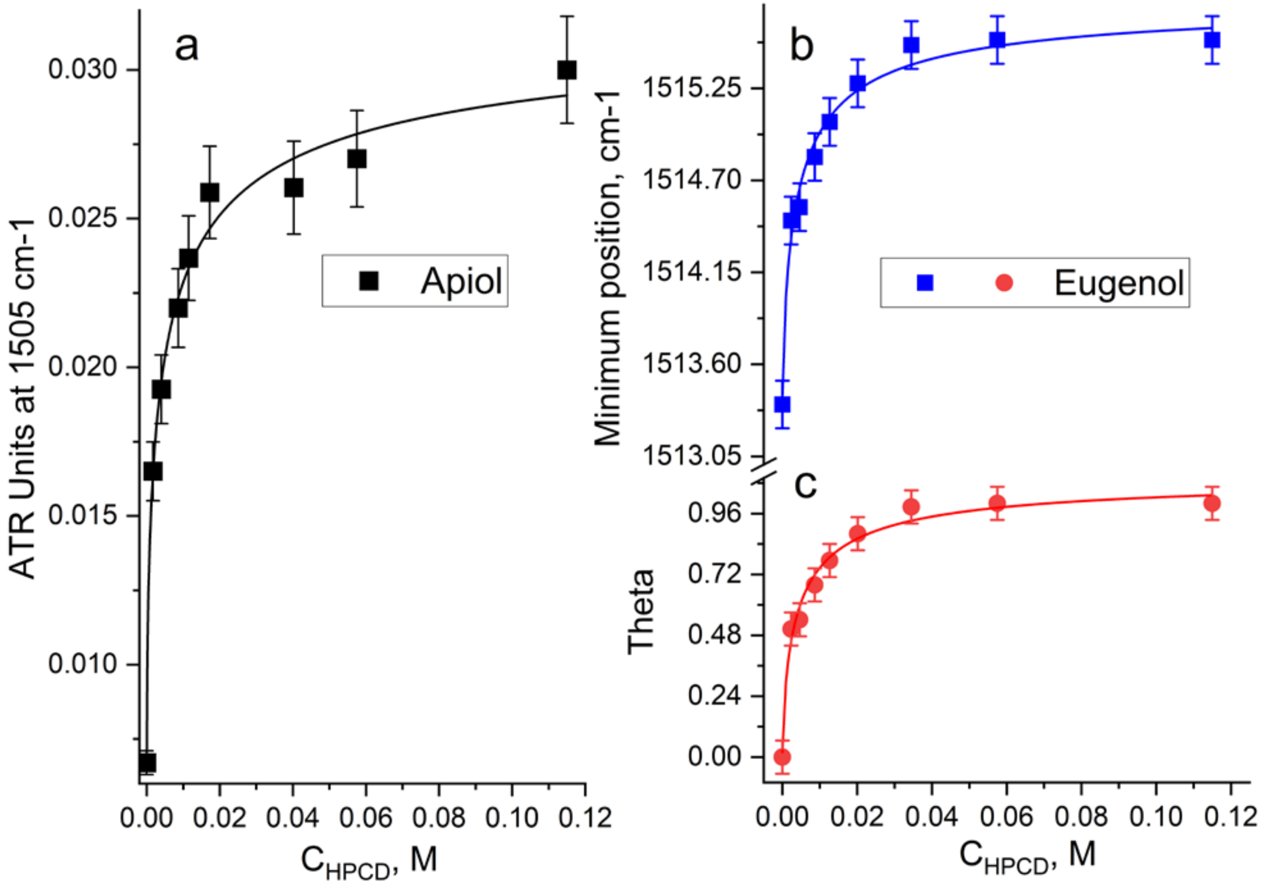

Figure S5. Second derivative (d^2A/dv^2) of FTIR spectra of eugenol (EG) and its inclusion complex with HPCD. A baseline correction was applied. Conditions are given in Materials and Methods, sections 3.4 and 3.5.

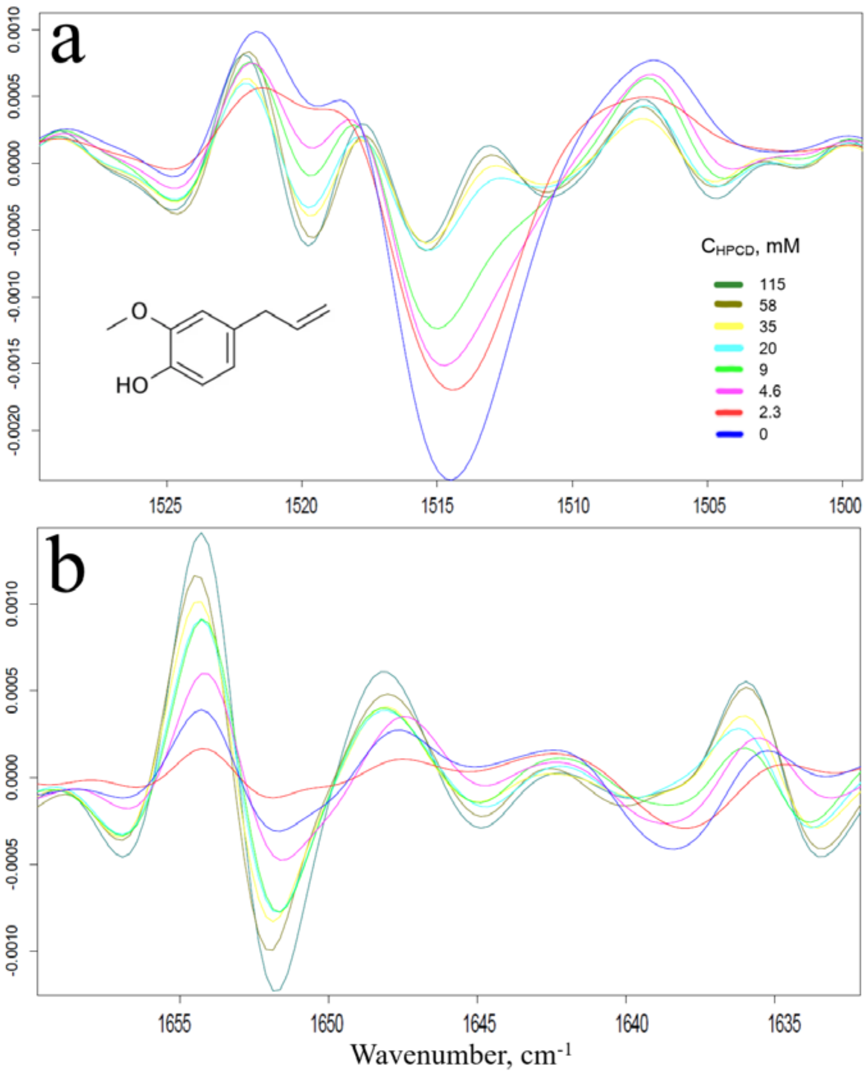

Figure S6. (a) Micrography of the EG-HPCD solid inclusion complex of 1.3 × 1.5 µm in size. (b), (c) Corresponding FTIR spectra at 16 points selected in (a). (d) Integral intensity of 1482–1562 cm^{-1} region in solid phase FTIR spectra of eugenol-HPCD. (e) Integral intensity of 1536–1499 cm^{-1} region in solid phase FTIR spectra, normalized to the corresponding intensity of C–O–C bonds of cyclodextrin at 1020-1180 cm^{-1}.

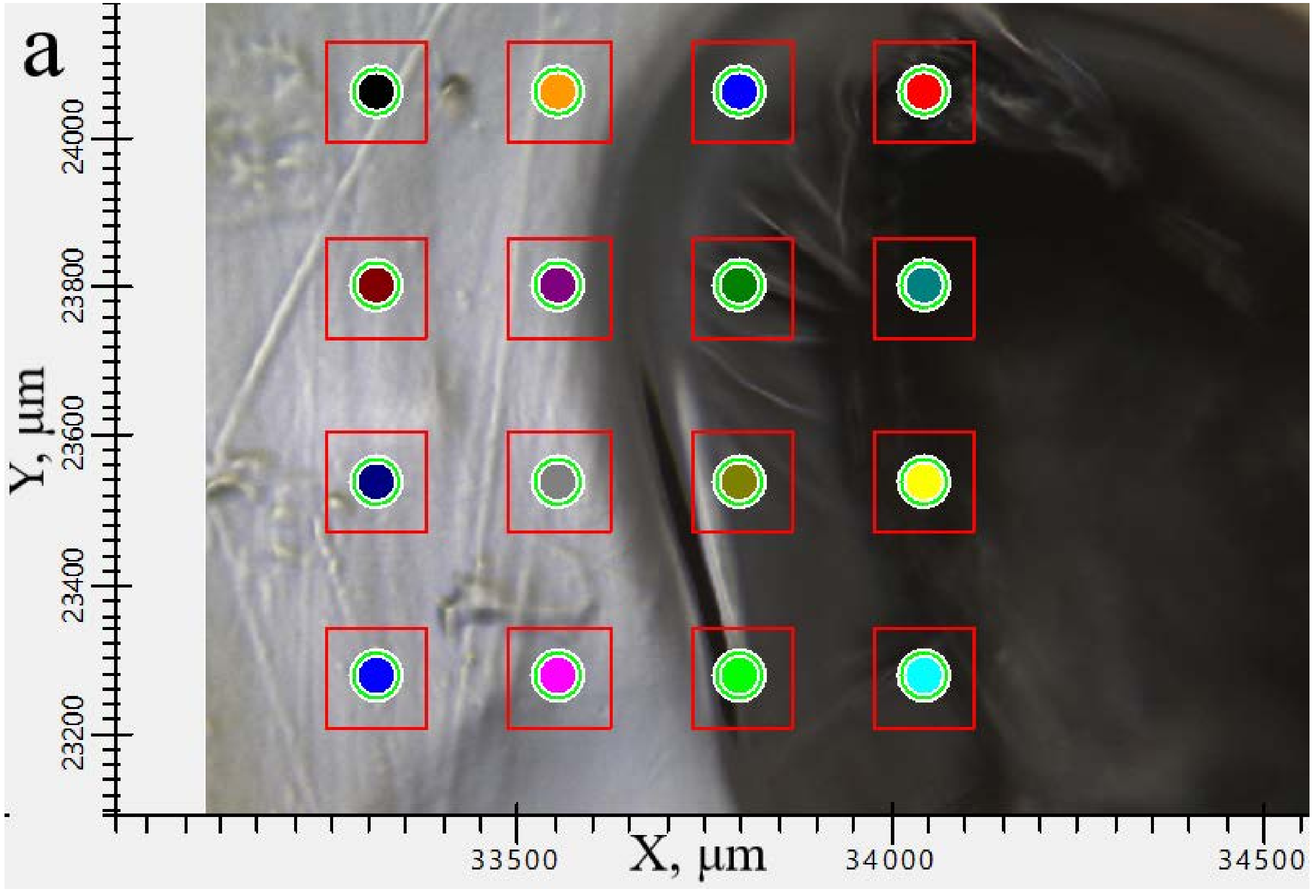

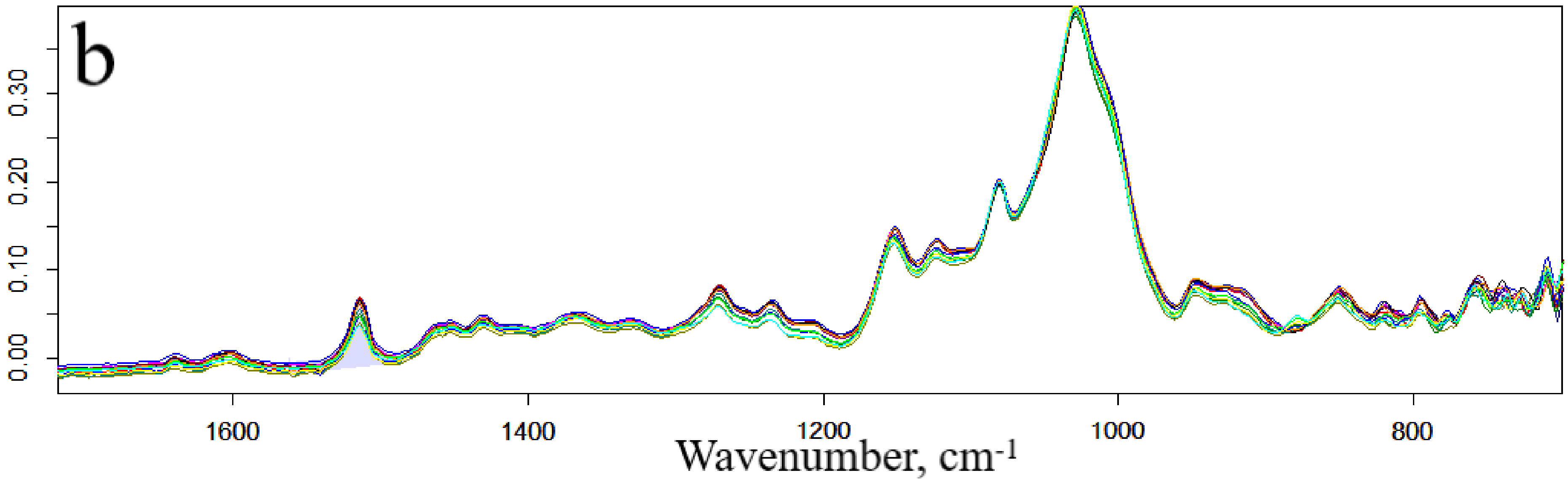
b
0.00
0.10
0.20
0.30
1600
1400
1200
1000
800
Wavenumber, cm-1

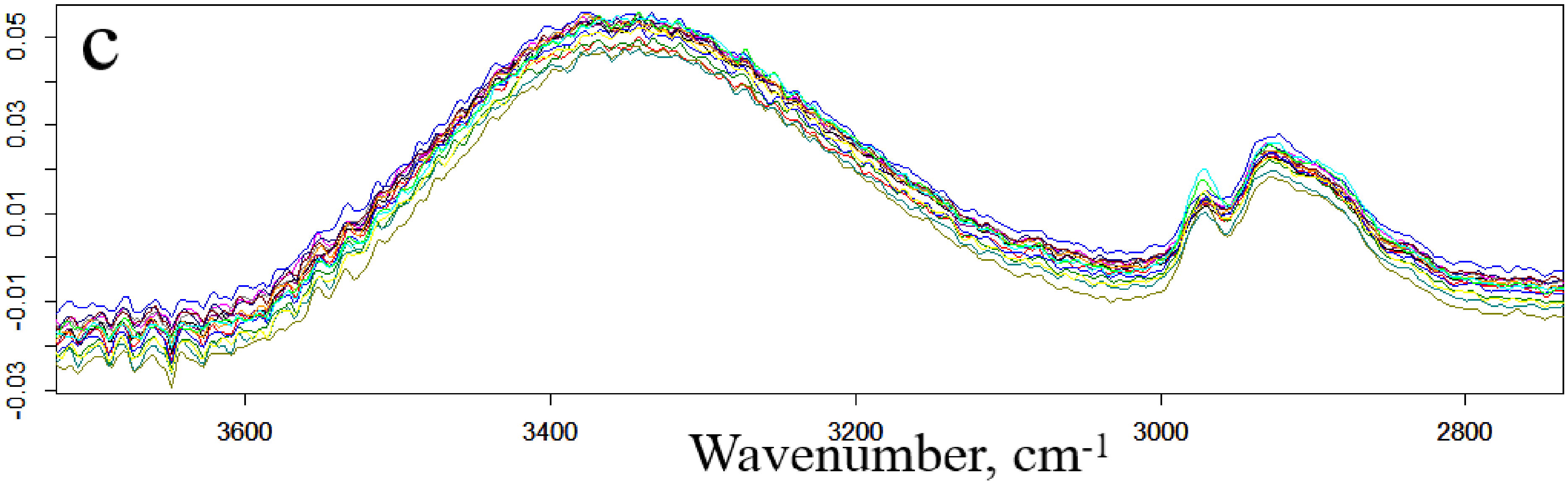
c
-0.03
-0.01
0.01
0.03
0.05
3600
3400
3200
3000
2800
Wavenumber, cm-1

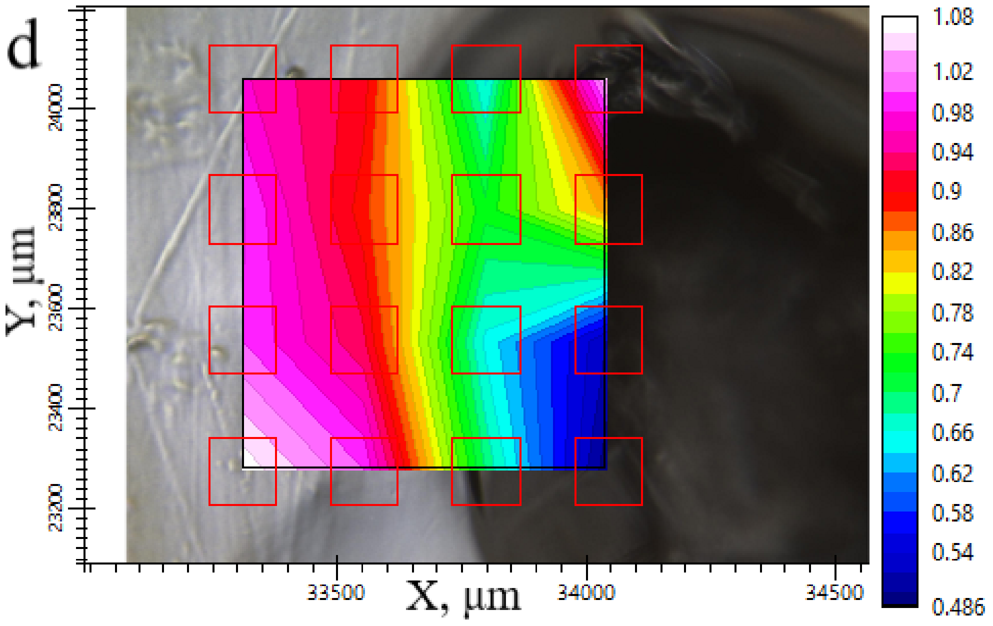
d
Y, µm
24000
23800
23600
23400
23200
X, µm
33500
34000
34500
1.08
1.02
0.98
0.94
0.9
0.86
0.82
0.78
0.74
0.7
0.66
0.62
0.58
0.54
0.486

e

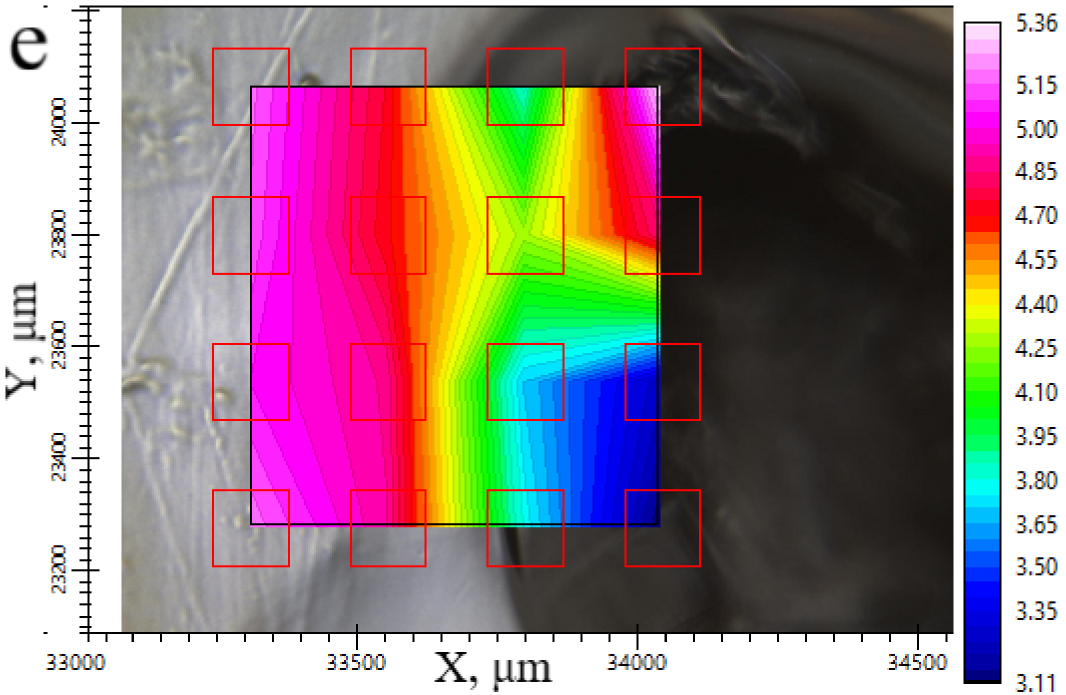
Y, µm
24000
23800
23600
23400
23200
33000
33500
34000
34500
X, µm
5.36
5.15
5.00
4.85
4.70
4.55
4.40
4.25
4.10
3.95
3.80
3.65
3.50
3.35
3.11

Figure S7. Micrographs illustrating the inclusion of eugenol (EG) in a HPCD. Dissolving oil droplets. (a) EG, (b) EG–HPCD (3.5:1), (c) EG–HPCD (1:1), (d) EG–HPCD (1:5). The molar ratio of the components is given in parentheses. Dissolving oil droplets of undissolved eugenol. Scale bar: 100 μm.

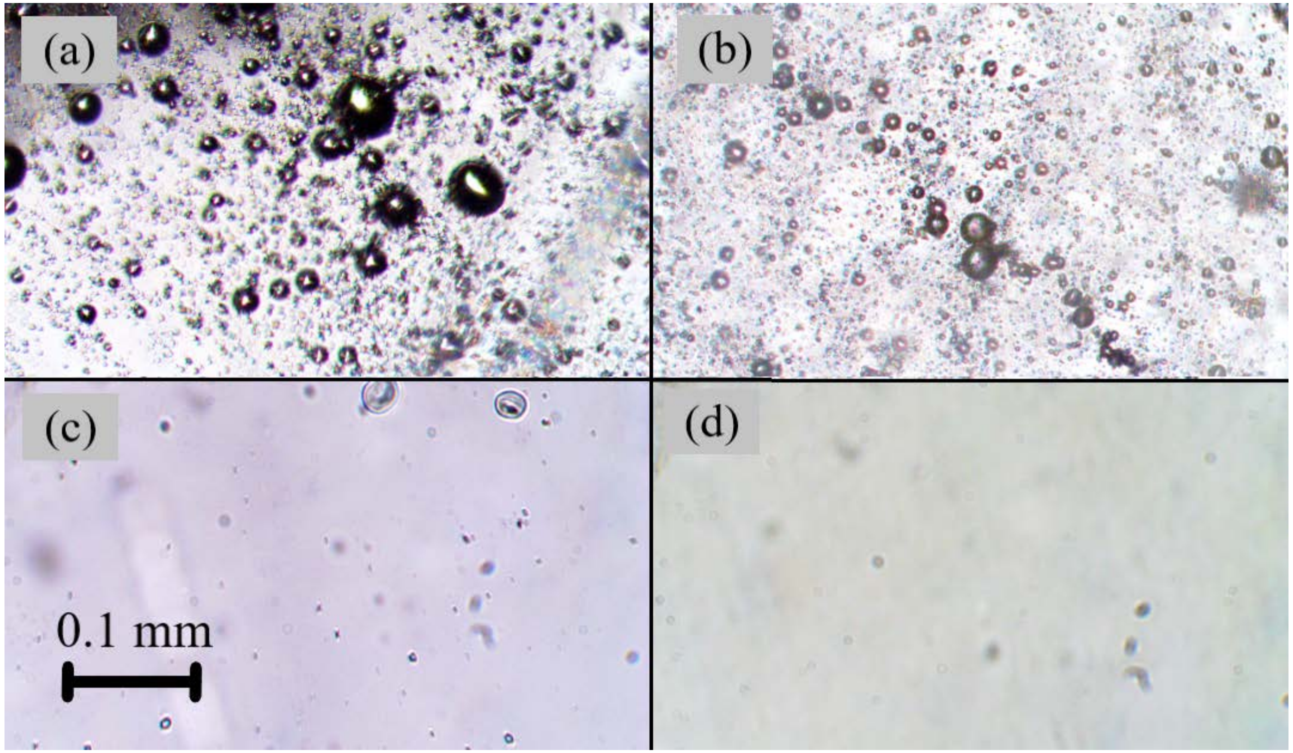

Figure S8. Micrographs illustrating the inclusion of apiol in a HPCD. Dissolving process. (a) Apiol, (b) Apiol–HPCD (2:1), (c) Apiol–HPCD (1:1), (d) Apiol–HPCD (1:15). The molar ratio of the components is given in parentheses. Scale bar: 100 μm.

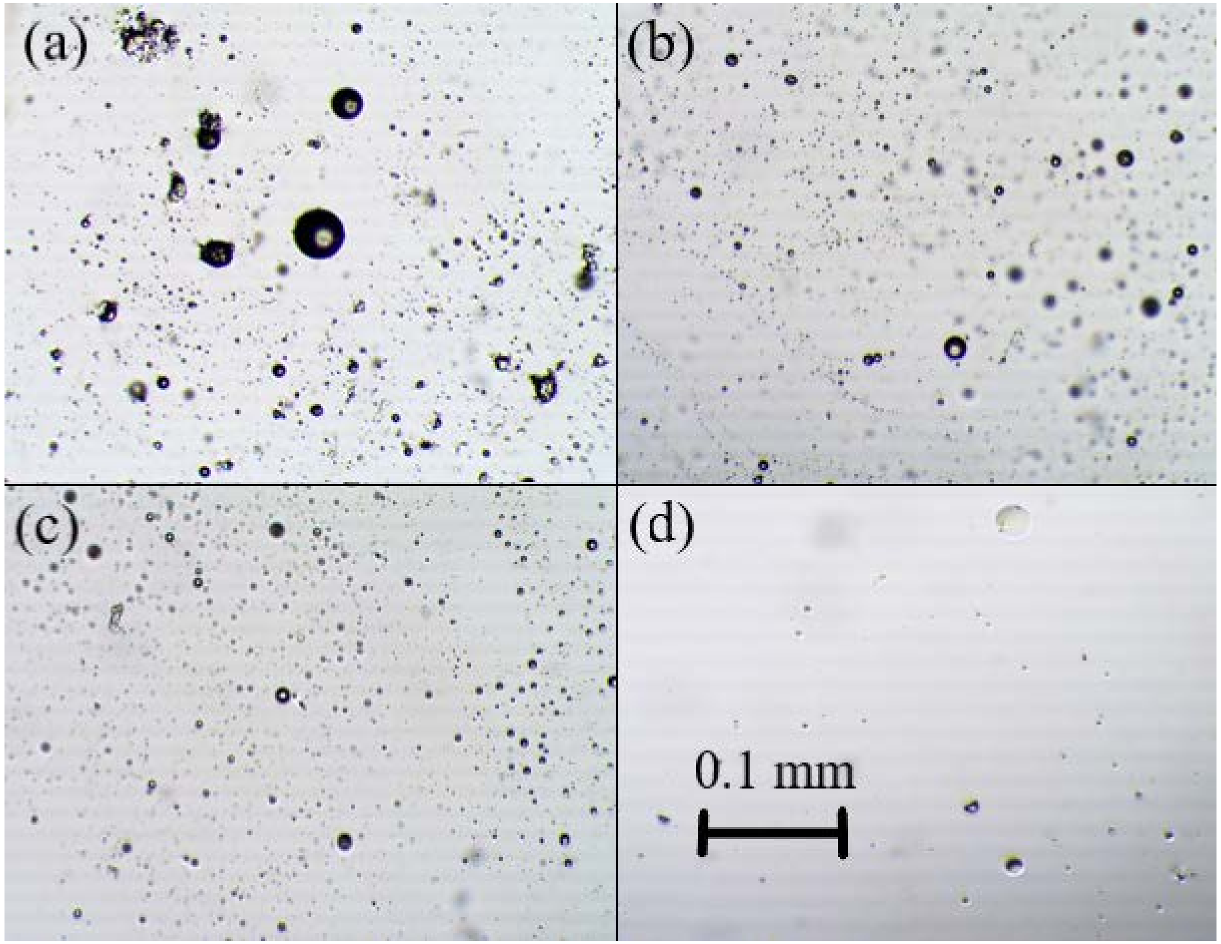

Figure S9. ^{1}H NMR-spectra (600 MHz) of apiol in DMSO-d6 (A); MCD-apiol in D_2O, molar ratio 2:1 (B); HPCD-EG in D_2O, molar ratio 2:1 (C); HPCD-Lev-EG in D_2O, molar ratio 4:1:1 (D). Computer simulation's structures of CD (E), MCD (F), HPCD (G) in comparison with literature X-ray data (H) [43].

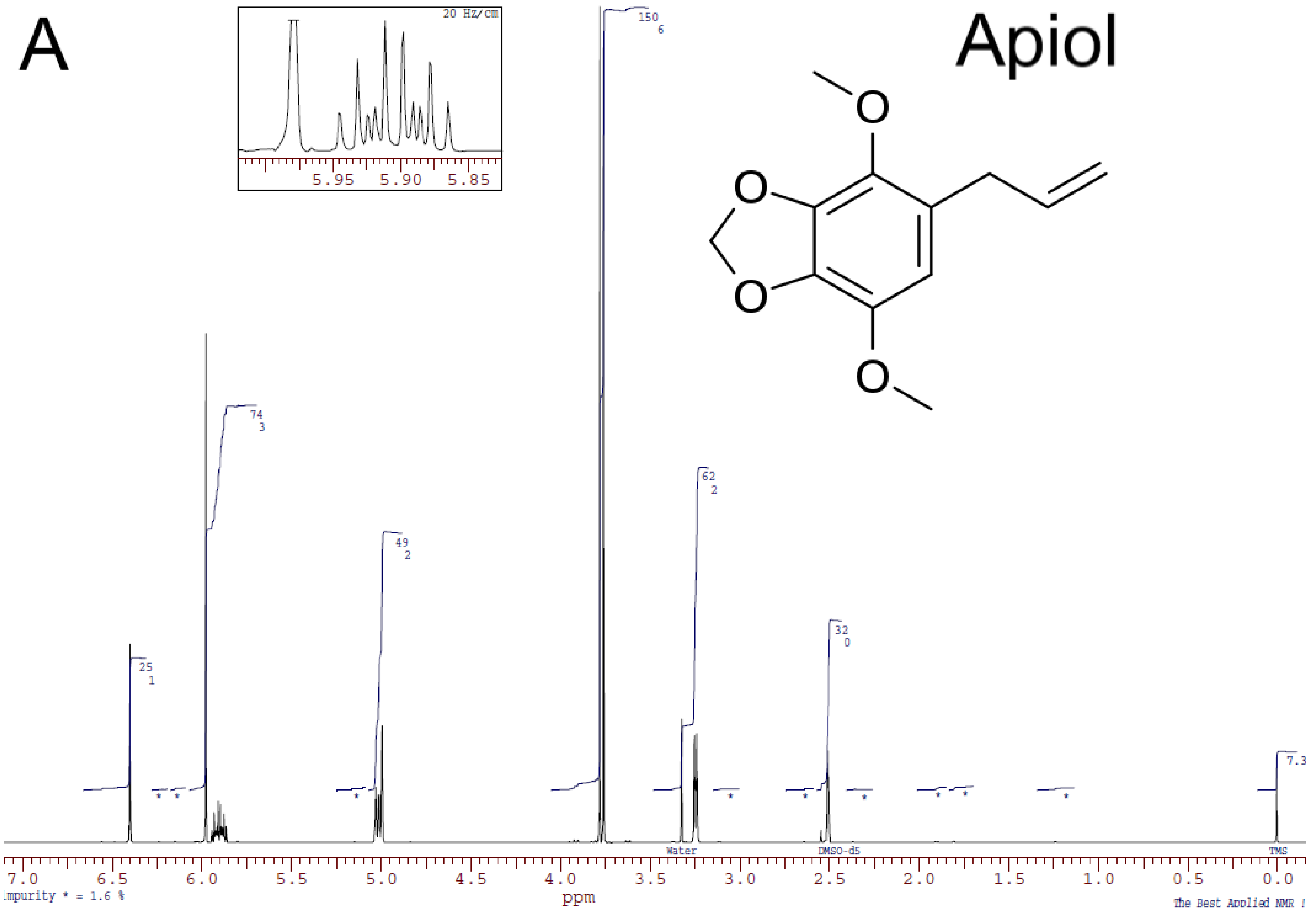

B Apiol-MCD

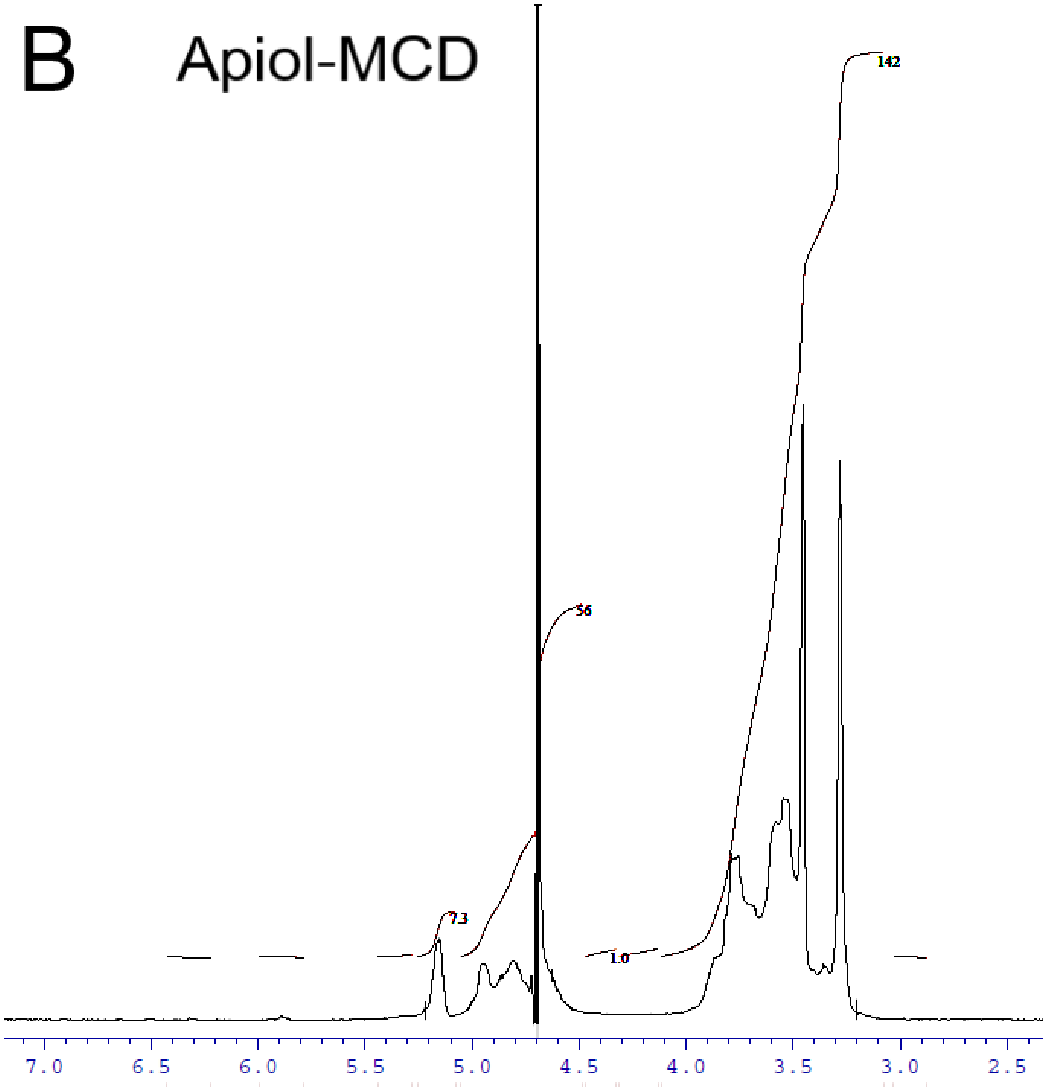

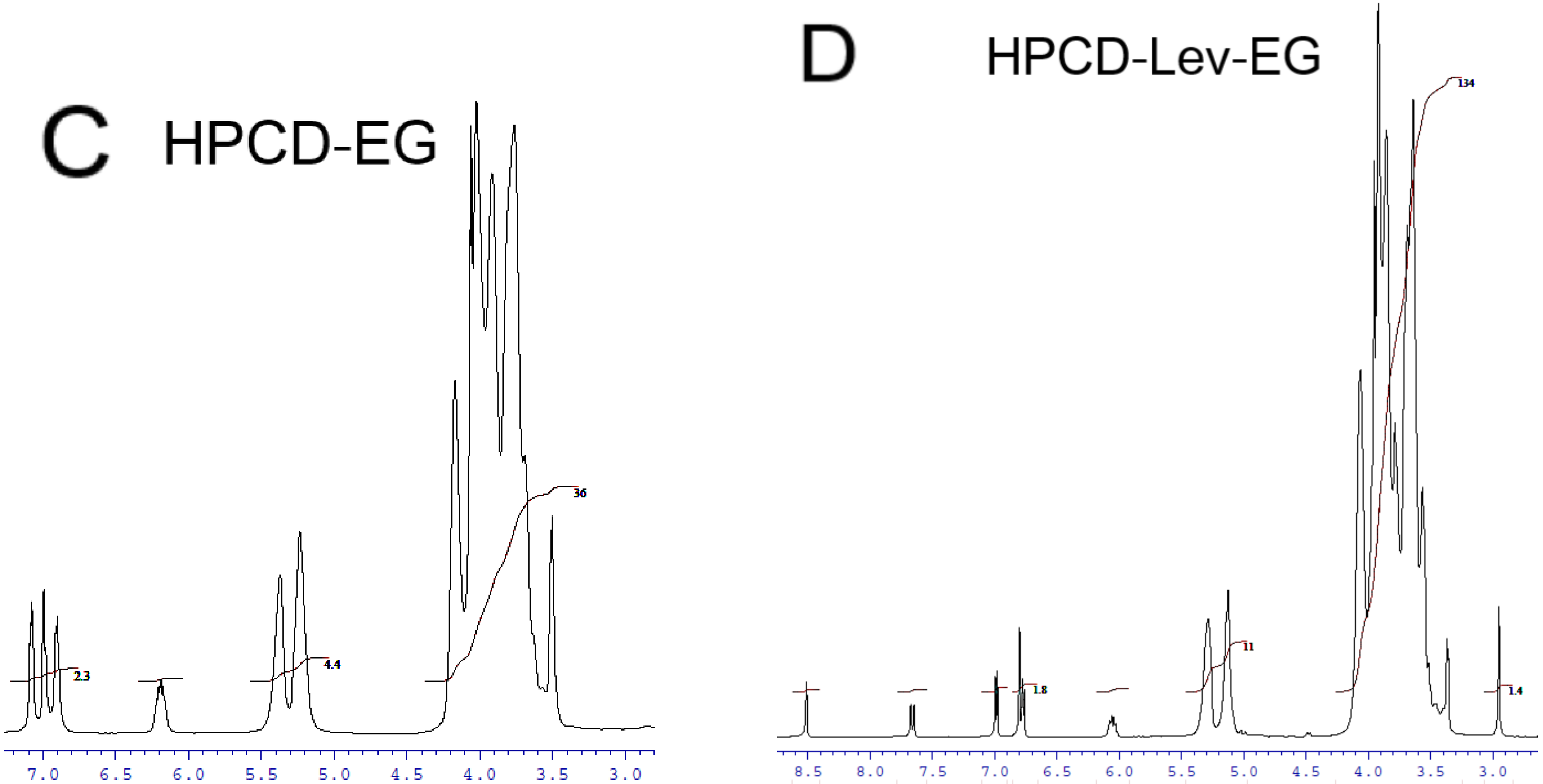

C
HPCD-EG
2.3
4.4
36
7.0
6.5
6.0
5.5
5.0
4.5
4.0
3.5
3.0
D
HPCD-Lev-EG
134
1.8
11
1.4
8.5
8.0
7.5
7.0
6.5
6.0
5.5
5.0
4.5
4.0
3.5
3.0

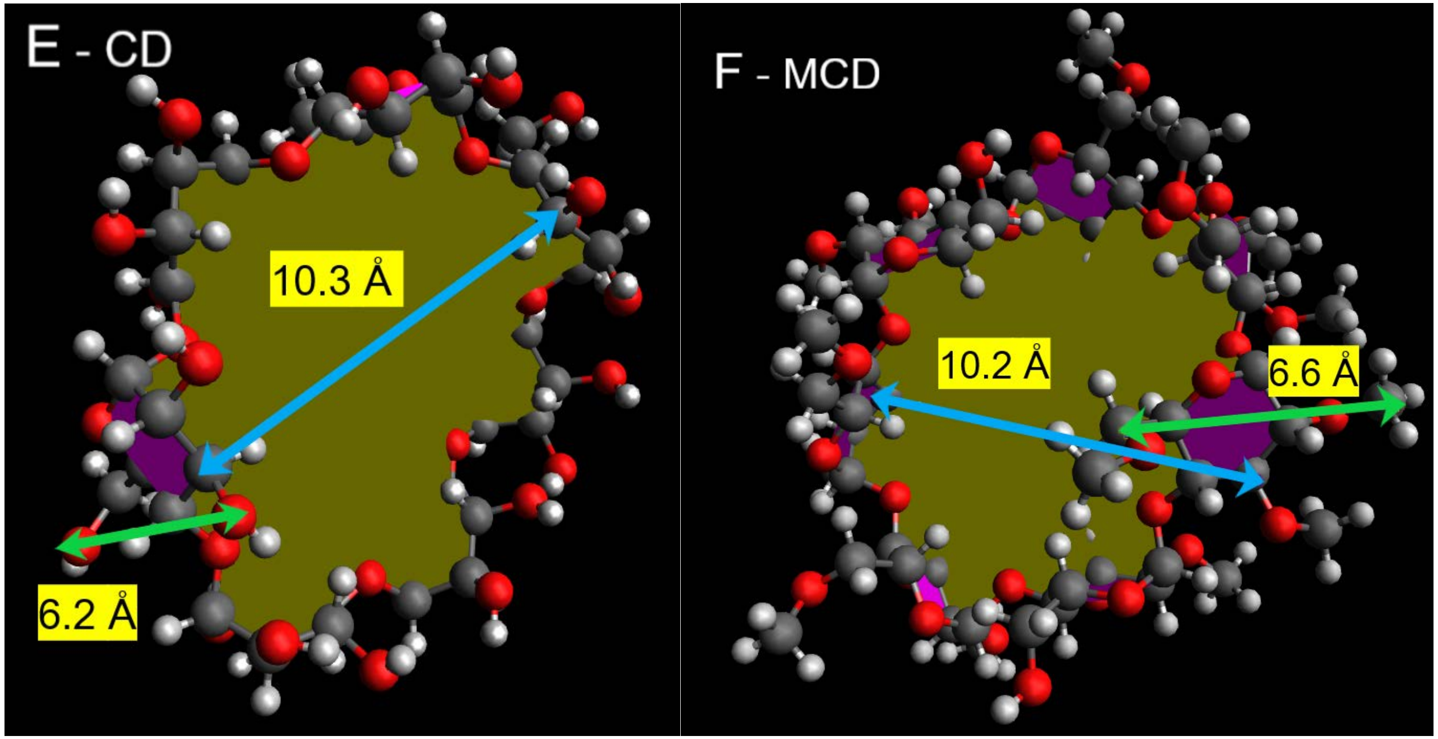
E - CD
10.3 Å
6.2 Å
F - MCD
10.2 Å
6.6 Å

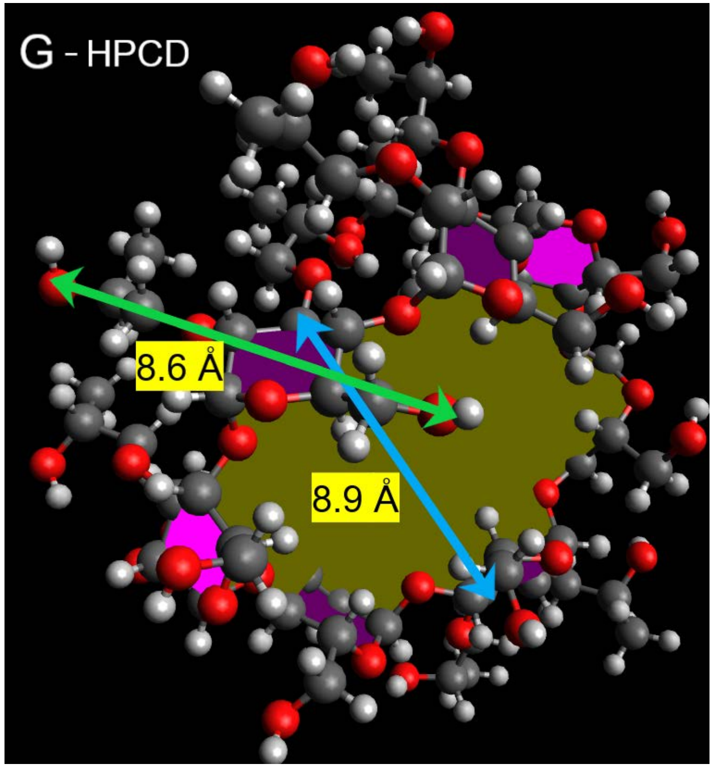
G - HPCD
8.6 Å
8.9 Å

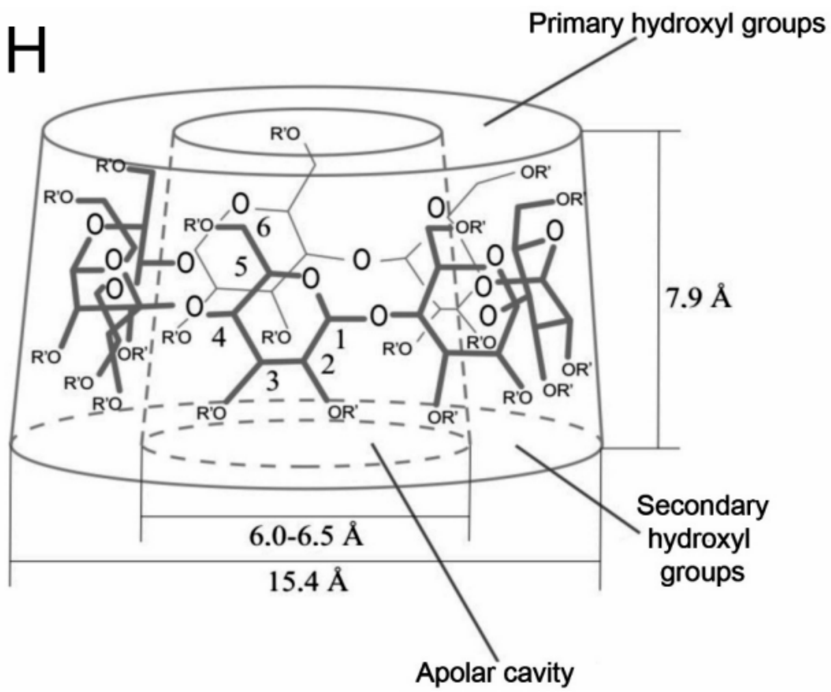
H
Primary hydroxyl groups
7.9 Å
Secondary hydroxyl groups
6.0-6.5 Å
15.4 Å
Apolar cavity

Figure S10. *B. subtilis* growth inhibition by (a) levofloxacin–MCD (1:2), (b) eugenol–MCD (1:2). The molar ratio of the components is given in parentheses. Small bold red crosses refer to the absence of bacterial growth inhibition; large green crosses indicate the diameter of growth inhibition zone. Experimental conditions here and thereafter in Figures S11–S14: pH 7.4 (0.01 M PBS), 37 °C, 24 h of incubation. MCD: methyl-β-cyclodextrin.

(a)

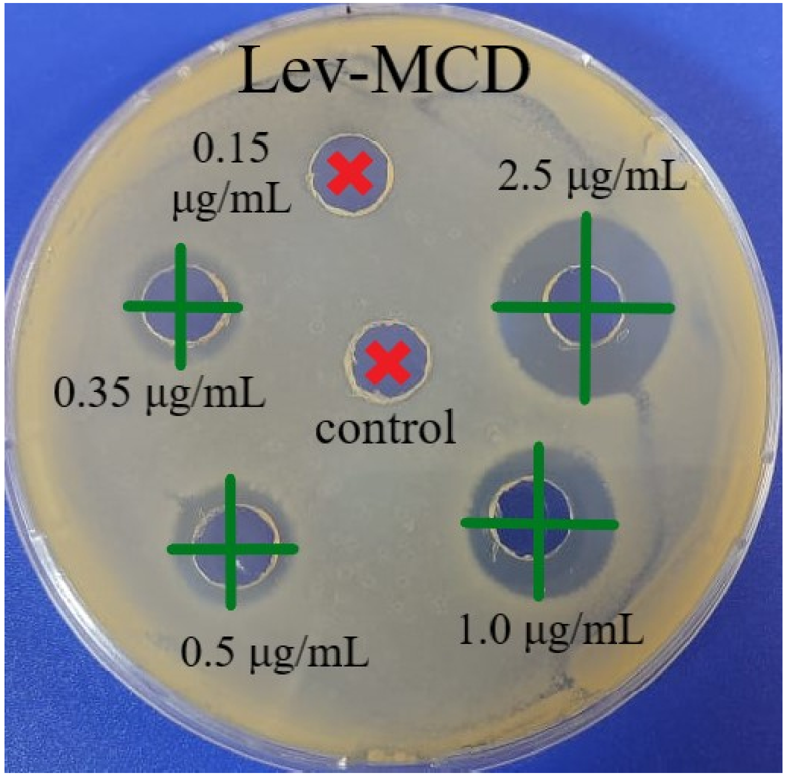

(b)

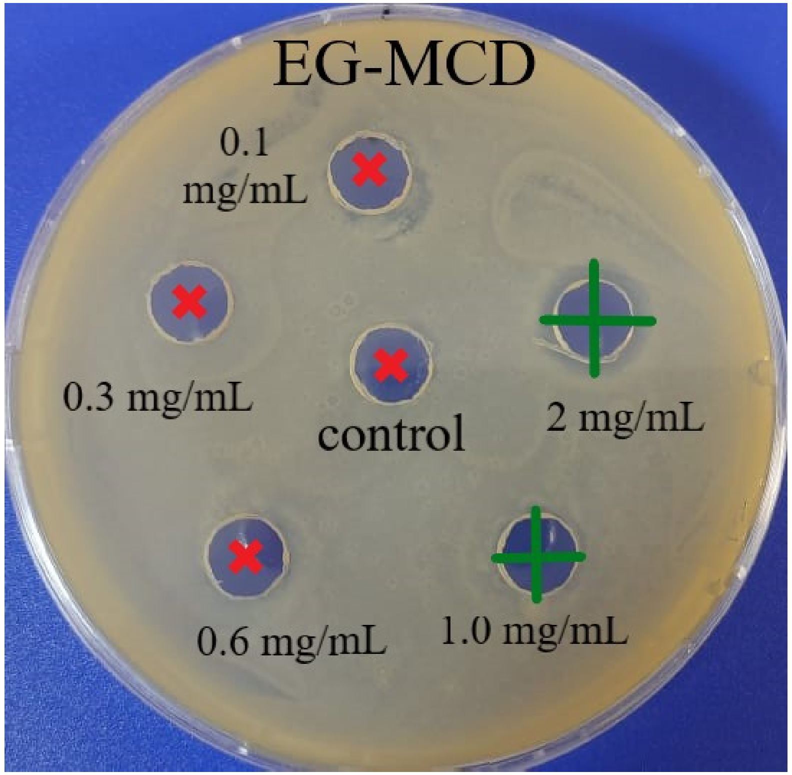

Figure S11. *E. coli* growth inhibition by levofloxacin–MCD (C(Lev) = 0.15 μg/mL) with adjuvant menthol–MCD (the concentrations of menthol are indicated in the figure). Small bold red crosses refer to the absence of bacterial growth inhibition; large red crosses indicate the diameter of growth inhibition zone.

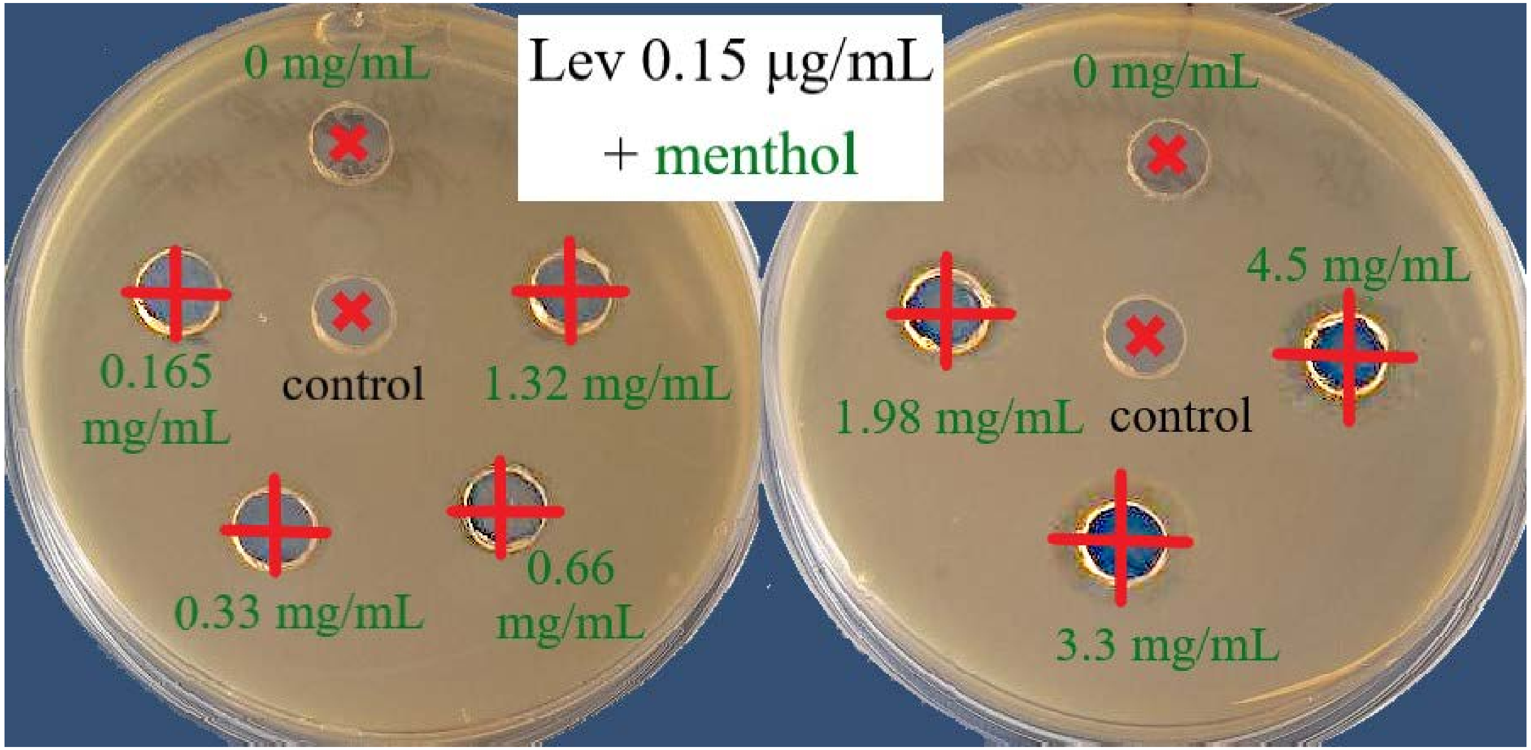

Figure S12. *E. coli* growth inhibition by levofloxacin–MCD (C(Lev) = 0.15 µg/mL) with adjuvant apiol–MCD (the concentrations of apiol are indicated in the figure). Small bold red crosses refer to the absence of bacterial growth inhibition. Black crosses indicate the diameter of growth inhibition zone.

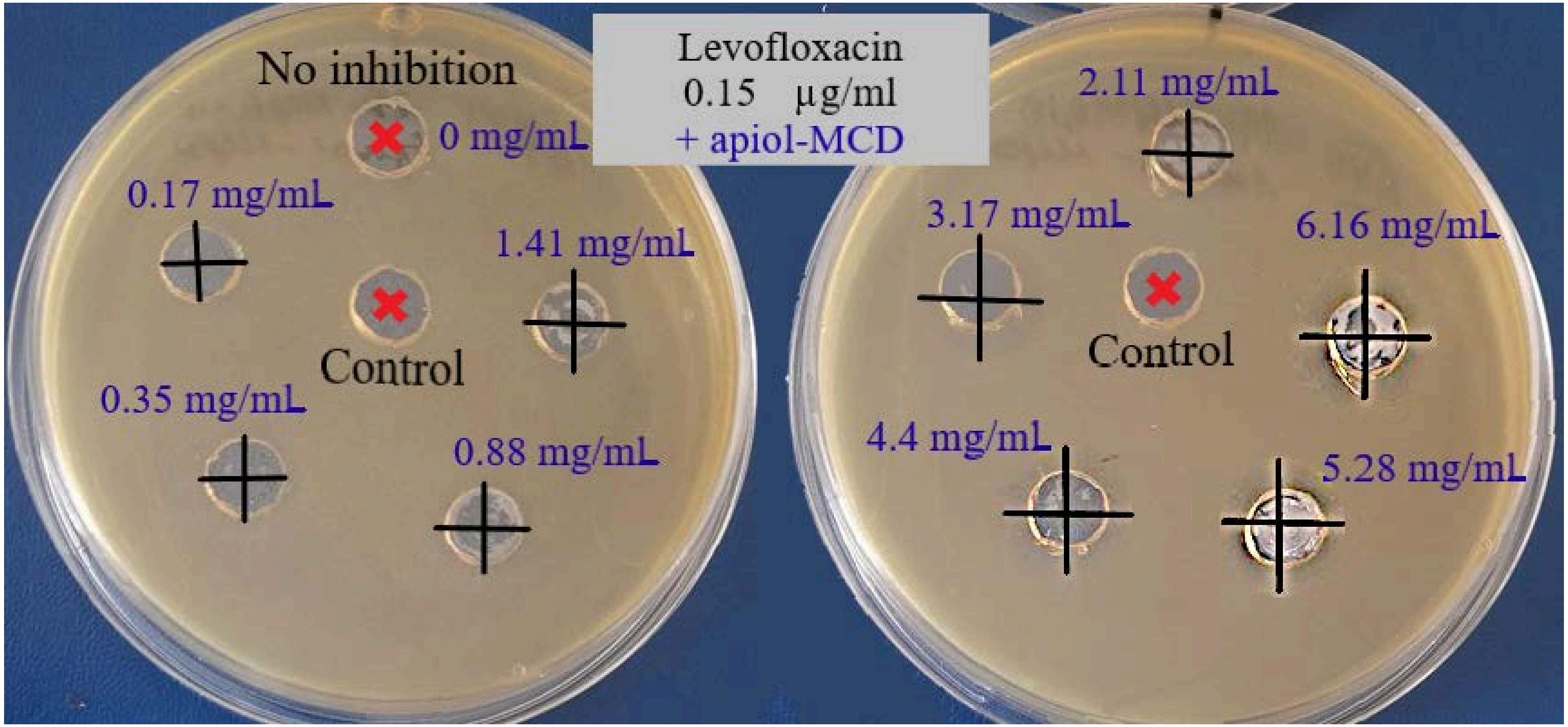

Figure S13. *B. subtilis* growth inhibition by levofloxacin–MCD (C(Lev) = 0.4; 0.7 and 1.5 μg/mL) with adjuvant eugenol–MCD (the concentrations of eugenol are indicated in the figure). Small bold red crosses refer to the absence of bacterial growth inhibition. Black crosses indicate the diameter of growth inhibition zone.

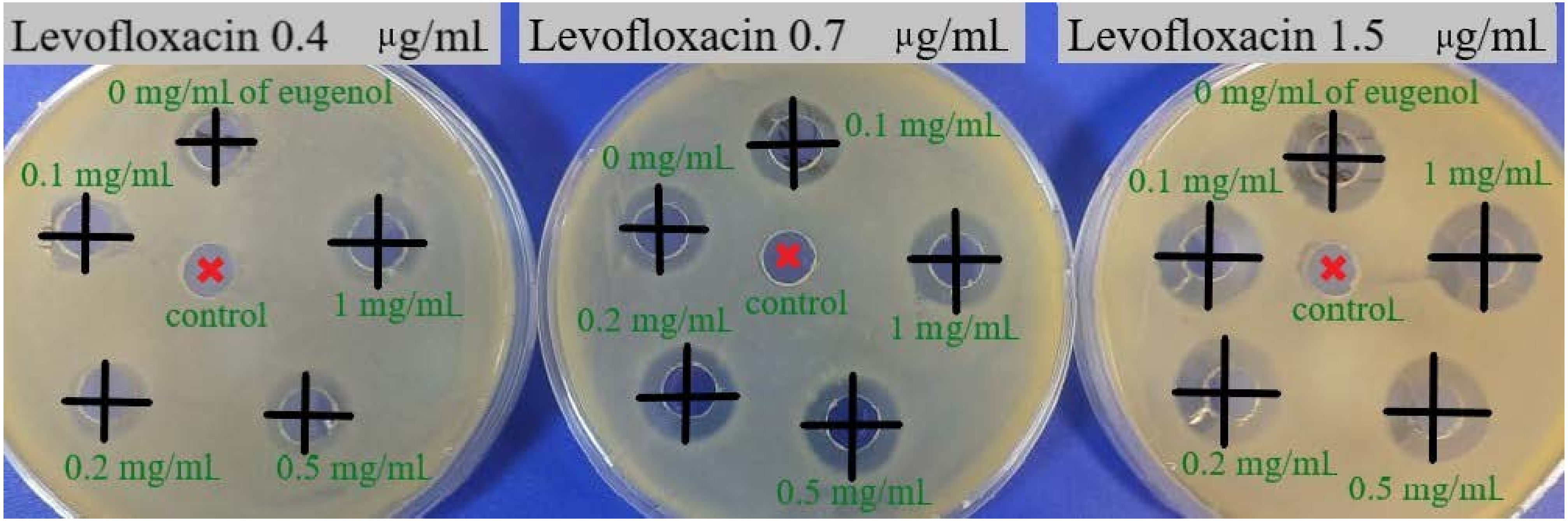

Figure S14. *B. subtilis* growth inhibition by levofloxacin–MCD (C(Lev) = 0.4; 0.7 and 1.5 μg/mL) with adjuvant safrole–MCD (the concentrations of safrole are indicated in the figure). Small bold red crosses refer to the absence of bacterial growth inhibition. Black crosses indicate the diameter of growth inhibition zone.

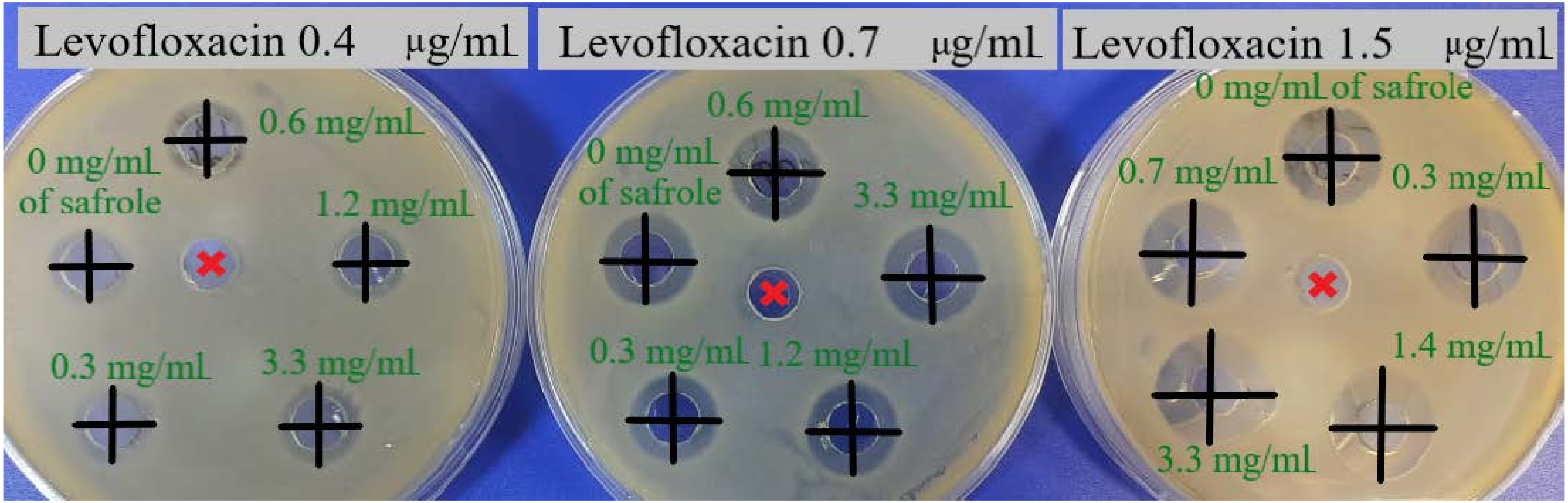

Supplement: Supplementary file 1 [file pharmaceuticals-15-00861-s001.zip › pharmaceuticals-1790570-supplementary.pdf]
